# The feasibility of age-specific travel restrictions during influenza pandemics

**DOI:** 10.1186/1742-4682-8-44

**Published:** 2011-11-11

**Authors:** Elson HY Lam, Benjamin J Cowling, Alex R Cook, Jessica YT Wong, Max SY Lau, Hiroshi Nishiura

**Affiliations:** 1 School of Public Health, The University of Hong Kong, Level 6, Core F, Cyberport 3, 100 Cyberport Road, Pokfulam, Hong Kong, People's Republic of China; 2 Saw Swee Hock School of Public Health, National University of Singapore, Singapore; 3 Department of Statistics and Applied Probability, National University of Singapore, 117546 Singapore; 4 Duke-NUS Graduate Medical School, 169857, Singapore; 5 PRESTO, Japan Science and Technology Agency, Honcho 4-1-8, Kawaguchi, Saitama, 332-0012, Japan

## Abstract

**Background:**

Epidemiological studies have shown that imposing travel restrictions to prevent or delay an influenza pandemic may not be feasible. To delay an epidemic substantially, an extremely high proportion of trips (~99%) would have to be restricted in a homogeneously mixing population. Influenza is, however, strongly influenced by age-dependent transmission dynamics, and the effectiveness of age-specific travel restrictions, such as the selective restriction of travel by children, has yet to be examined.

**Methods:**

A simple stochastic model was developed to describe the importation of infectious cases into a population and to model local chains of transmission seeded by imported cases. The probability of a local epidemic, and the time period until a major epidemic takes off, were used as outcome measures, and travel restriction policies in which children or adults were preferentially restricted were compared to age-blind restriction policies using an age-dependent next generation matrix parameterized for influenza H1N1-2009.

**Results:**

Restricting children from travelling would yield greater reductions to the short-term risk of the epidemic being established locally than other policy options considered, and potentially could delay an epidemic for a few weeks. However, given a scenario with a total of 500 imported cases over a period of a few months, a substantial reduction in the probability of an epidemic in this time period is possible only if the transmission potential were low and assortativity (i.e. the proportion of contacts within-group) were unrealistically high. In all other scenarios considered, age-structured travel restrictions would not prevent an epidemic and would not delay the epidemic for longer than a few weeks.

**Conclusions:**

Selectively restricting children from traveling overseas during a pandemic may potentially delay its arrival for a few weeks, depending on the characteristics of the pandemic strain, but could have less of an impact on the economy compared to restricting adult travelers. However, as long as adults have at least a moderate potential to trigger an epidemic, selectively restricting the higher risk group (children) may not be a practical option to delay the arrival of an epidemic substantially.

## Background

Long-distance international flights facilitate human movement, enhancing not only cross-border travel but also the global spread of infectious diseases. The well-connected global airline network allows multiple importations of infected individuals and rapid dissemination of an epidemic to a previously disease-free country [1], as was observed during the influenza H1N1-2009 pandemic [2-4]. Border controls aim to identify and restrict movement of infected and/or infectious individuals at the border, thereby lessening the untraced importation of infection from a source country. One such border control measure is to impose restrictions on travel that radically cut traveler numbers, a potentially effective option during the early stage of a pandemic. Perhaps because a large-scale restriction policy presents political and economic difficulties to our highly connected global society, long-lasting and large-scale mandatory restriction did not take place during the H1N1-2009 pandemic.

Prior to the H1N1-2009 pandemic, several studies assessed the effectiveness of travel restrictions [5-10], but none was equivocal in supporting it to be used, for two major reasons. First, if the epidemic is already established overseas, there will be a continuous exportation of cases, which will eventually allow the epidemic to establish a foothold in the country in question regardless of the presence of control efforts. As a result, travel restrictions could only delay the arrival of the epidemic, perhaps for weeks or months [7]. Second, even though travel restrictions may effect a delay, several published studies agree that the epidemic can only be delayed substantially if an implausibly high (~99%) proportion of trips are prevented [8-10]. The public health effort that is required to restrict 99% of travelers may not be too different from that of completely shutting down the border, and such an extreme restriction may not be feasible due to its impact on world trade and economic activity. In addition, the benefit, i.e. a brief delay before widespread community transmission, might not warrant the costs of travel restrictions [11]. The revised International Health Regulations, issued by the World Health Organization in 2005, emphasizes the need to avoid unnecessary interference with international traffic and trade [12]. The combination of scientific evidence of poor effectiveness, the large prospective economic impact, and international law have thus made policies that impose blanket travel restriction policies unfeasible.

Epidemiological studies of the influenza H1N1-2009 pandemic have revealed that transmission was highly heterogeneous and mainly maintained by school-age children [13-17]. However, international travel is usually dominated by adults, and consequently imported cases have also been dominated by adults [18]. In a previous study [19], a multivariate stochastic model was employed to examine the age-related impact of imported cases on the establishment of a major epidemic, which suggested that the predominance of adult travelers might delay the arrival of an epidemic with the same characteristics as the 2009 pandemic. This combination of age-assortative mixing, more infection among children, and greater volume of travel among adults raises the question: how effective would *selective *age-specific travel restrictions, that target child travelers, be? Although restricting adult international travel may be economically damaging, the impact of preventing child travel, by cancelling school trips for instance, is likely to be less severe. The purpose of the present study is to examine the potential effectiveness of age-specific selective travel restriction against an influenza pandemic with similar characteristics to that of the 2009 pandemic, using a parsimonious statistical model.

## Methods

### Theoretical basis and hypothetical settings

Empirical observations of the H1N1-2009 pandemic support the hypothesis that age-specific travel restrictions could be effective. Table 1 lists countries that reported initial imported cases in children. Although it is known that adults were predominant among imported cases, children were also among the first identified imported cases in many countries. Table 2 shows countries in which initial or almost initial local cases were observed among children (e.g. school outbreaks). Undetected imported infections in children may have fueled some of the initial school outbreaks, and again the restriction of child movement may have the potential to prevent those clusters. These observations justify our motivation to investigate the effectiveness of child movement restrictions in reducing the risk of an epidemic and delaying an epidemic.

**Table 1:** Countries initially reporting child imported cases during influenza pandemic (H1N1-2009)

Country	Report month	Descriptions
Australia [36]	May 2009	The first confirmed Victorian case was reported in a child returning from USA
Argentina [37]	March 2010	First case detected in Chile's Quake-hit area was a 5-year old child
Brazil [38]	May 2009	The first four imported cases were found in young adults who had travelled to Mexico and the USA
China [39]	May 2009	The first imported case was a student returning from Canada. The second and third imported cases were notified in students coming from USA
Ecuador [40]	May 2009	First case of H1N1-2009 was a student aged 13 returning from the USA
France [41]	May 2009	Second imported case was a student aged 17 from Mexico
Italy [42]	May 2009	A 11-year-old male child and a 33-month-old infant were confirmed to be the first and third cases of H1N1-2009 in Rome
Japan [43]	May 2009	Three teenage students and a teacher were confirmed to be the first four imported H1N1-2009 cases after returning from a school trip in Canada
New Zealand [44]	April 2009	The first imported cases in New Zealand arrived in a group of students returning from a visit to Mexico
Portugal [45]	June 2009	Third imported case was a 8-year-old child returning from Toronto
Singapore [46]	June 2009	Eighth case is a 15-year-old Singaporean male who travelled from India to Orlando and Atlanta
Spain [47]	July 2009	13 cases of influenza evacuated from a camp in La Vera. Of the 13 cases, 11 were children
Thailand [48]	May 2009	First imported case was a 17-year-old Thai female student returning from Mexico
United Kingdom [49]	April 2009	First confirmed case, a pupil at a school in England, was imported
United States of America [50]	April 2009	First two cases were identified in two children in California

**Table 2:** Countries with early child clusters of cases (or school outbreaks) during the influenza pandemic (H1N1-2009)

Country	Report month	Descriptions
Australia [51]	February 2010	The return of children to school in the North American autumn 2009 was associated with a substantial increase in the number of cases of pandemic H1N1 2009 influenza
Australia [52]	May 2009	55% of H1N1-2009 cases in Australia and 63% of cases in Victoria to date have been school aged children (5 - 17 years)
Argentina [53]	May 2009	First imported case seeded an elementary school outbreak in Buenos Aires, and, within days, several schools reported increasing numbers of cases
Cyprus [54]	June 2009	The disease spread quickly, initially among younger people who visited tourist resorts and entertainment clubs or school-aged children who stayed at camping places or summer schools
France [55]	July 2009	The first time in France, a confirmed outbreak without history of travel occurred in a secondary school in Toulouse district
Germany [56]	June 2009	About two thirds of indigenous cases were associated with two large school-associated outbreaks
Italy [42]	December 2009	First cluster of in-country transmission involved a 33-month-old and a 11-year-old child
Japan [57]	May 2009	Most of new cases were seen in high school students in western Japan
Macau [58]	July 2009	Three locally-infected cases were all local primary school students
Malaysia [59]	July 2009	The first case was a student returning from the US followed by multiple clusters in schools, which all involved cases returning from abroad with the infection.
Thailand [48]	October 2009	The number of reported cases was most prevalent in primary school students aged 6-12 years, followed by secondary school students aged 13-18 years
United Kingdom [49]	August 2009	First confirmed case, a pupil at a school in England, was imported. During the following two weeks, 16 further cases were confirmed with epidemiological links to the first imported case.
United States of America [60]	October 2009	In May 2009, one of the earliest outbreaks of 2009 pandemic influenza A virus (pH1N1) infection resulted in the closure of a semi-rural Pennsylvania elementary school

Employing a simple statistical model, we assess the effectiveness of travel reduction during the first 50 days of a pandemic, roughly corresponding to the time period in which enhanced surveillance was conducted in 2009 (e.g. in Japan [14,20]). We consider a case study modeled on the H1N1-2009 pandemic in Hong Kong, a special administrative region of the People's Republic of China, during the early epidemic period. From 1 May to 19 July 2009, there were 113 confirmed imported cases in Hong Kong [19] with a daily average of 2 cases. Assuming that approximately 30% of infected travelers were identified [21], and ignoring the initial linear increase in the rate of growth number of new imported cases for the first 50 days coupled with a sampling of infected travelers from local cases, we expect a daily average of 10 imported cases. Since we consider a 50 day time horizon, this extrapolates to 500 imported cases in this hypothetical scenario. Given that more than 60% of confirmed cases were adults [18], we assume that children and adults comprise of 30% and 70% of the total of imported cases, respectively. In other words, everyday there are *n*_c _= 3 child cases and *n*_a _= 7 adult cases imported, and over 50 days, there would be *N*_c _= 150 child and *N*_a _= 350 adult imported cases. It should be noted that the proportion of children among all international travelers is about 10% (e.g. those aged below 20 accounted for 10.4% of all legal immigrations in Japan, 2010 [22]), that is, less than the proportion of imported child cases. This indicates that, under our hypothetical scenario with stationary importation, the risk of influenza among child travelers is approximately fourfold that of adult travelers (i.e. (0.3/0.1)×(0.9/0.7) = 3.86), and also that restricting child travel is 4 times more likely to prevent an entry of influenza case than restricting adult travel. Connecting flights from Mexico City to Hong Kong take longer than 27 hours, and considering additional times from home/hotel to airport, we assume that a time lag of *δ *= 2 days from infection until the case starts influencing local transmission in Hong Kong.

### Modelling methods

We use two outcome measures to quantify the effectiveness of selective travel restrictions. The first is the probability of an epidemic, defined as the probability of observing a *major *epidemic, that is, an epidemic not going extinct by chance [23]. The second uses the time required for a major epidemic to take place, conditional on non-extinction in the short term, to quantify the delay effect, i.e., the difference in the timing of epidemic overshoot between scenarios in the presence and absence of selective travel restrictions.

To model age-dependent transmission dynamics, we employ the two-by-two next generation matrix **K **which describes within- and between-group transmissions in a population that consists of children and adults. Throughout this article, we label children as type *c *and adults as type *a*. Let *R*_ij _denote the average number of secondary cases in hosts of type *i *generated by a single infected individual of host type *j*. We assume the offspring distribution to be Poisson, and adopt a previously studied parametric form ([13,19]), i.e.

(1) $$R_{ij}\propto \left\{\begin{array}{c} \left(1-\theta \right)\alpha_{i}\beta_{j}q_{i}\;for\;i\neq j,\\ \theta \alpha_{i}\beta_{j}+\left(1-\theta \right)\alpha_{i}\beta_{j}q_{i}\;\;for\;i=j, \end{array}\right)$$

where *q*_i _is the relative size of the subpopulation *i *(i.e., *q*_c_+*q*_a _= 1), α_i _and β_j _represent age-specific susceptibility and infectiousness of hosts of type *i *and *j *respectively, while θ is an assortativity coefficient representing the proportion of contacts reserved for within-group mixing (and (1-θ) represents the proportion of contacts subject to random or proportionate mixing). As the baseline setting, parameters are fixed at *q*_c _= 0.32, α_c _= 2.06, α_a_=β_c_=β_a _= 1, and θ = 0.50 [13]. The dominant eigenvalue of **K **gives the reproduction number *R*. Given that empirical estimates of *R *for H1N1-2009 ranged from 1.2 to 2.3 by limiting the choice of mean generation time [24], we examine three different values of the reproduction number, 1.2, 1.6 and 2.0 to account for relevant uncertainty in the transmission potential of a pandemic. For each *R*, we rescale the next generation matrix by

(2) $$M=\frac{R}{\rho \left(K\right)}K$$

where ρ(.) denotes the dominant eigenvalue. We use the rescaled **M **for all calculations.

### The risk of an outbreak under targeted travel restrictions

We follow a previous study [19], adopting linear approximation of the growth of cases during the early phase of an epidemic. That is, we calculate the probabilities π_c _and π_a _of extinction given a single infected child or adult, respectively, by iteratively solving the following equations:

(3) $$\begin{array}{c} \pi_{c}=\frac{1}{1+R_{cc}\left(1-\pi_{c}\right)+R_{ac}\left(1-\pi_{a}\right)},\\ \pi_{a}=\frac{1}{1+R_{ca}\left(1-\pi_{c}\right)+R_{aa}\left(1-\pi_{a}\right)}. \end{array}$$

The solution rests on a homogeneous birth-and-death process, and its derivation can be found elsewhere [25]. We explicitly account for the delay in infection-age among imported cases [3], that is, that imported cases must have been infected prior to arrival in Hong Kong. A slight delay, even of a fraction of a day, is not negligible in the natural history of an acute illness such as influenza [26]. For simplicity, we assume that there was a constant delay of *δ *= 2 days between infection and arrival for all imported cases. Let *k*_c _and *k*_a _be the number of imported infections among children and adults, respectively, in Hong Kong that are capable of causing on secondary transmission. To facilitate the calculation of the probability of extinction above, we assume that the generation time of influenza is exponentially distributed with mean 1/*γ *= 3 days [19]. Given *N*_c _and *N*_a _child and adult imported cases, the probability that *m*_c _and *m*_a _local index cases will result is

(4) Pr(mj=s)= ∑ka=0Na∑kc=0NcNckc exp-γkcδ1- exp-γδNc-kc×Naka exp-γkaδ1- exp-γδNa-ka×kc exp-γδRjc+ka exp-γδRjas×exp-kc exp-γδRjc-ka exp-γδRjas!

for *j*= *c *or *a *[9]. The first two binomial terms account for the probability that child and adult imported cases arrive in Hong Kong while still infectious, while the last, Poisson, term describes the probability that those infectious imported cases generate *m*_c _(or *m*_a_) local index cases. As a result, the probability of an epidemic is

(5) $$\mathrm{Pr}\left(epidemic\right)=1-\overset{\infty }{\underset{s_{c}=0}{\sum }}\mathrm{Pr}\left(m_{c}=s_{c}\right)\overset{s_{c}}{\underset{c}{\pi }}\overset{\infty }{\underset{s_{a}=0}{\sum }}\mathrm{Pr}\left(m_{a}=s_{a}\right)\overset{s_{a}}{\underset{a}{\pi }}.$$

By varying the total number of child and adult travelers *N*_c _and *N*_a _as part of a simulated travel restriction policy, we examined the effectiveness of travel restriction in reducing the epidemic risk.

### Time taken for a local epidemic to occur under targeted travel restrictions

Replacing the total numbers of child and adult imported cases for the first 50 days (i.e. *N*_c _and *N*_a_) in (4) by the daily numbers of child and adult imported cases (i.e. *n*_c _and *n*_a _where *n*_c_+*n*_a _= 10), we obtain the daily probability (*p*) that an epidemic is initiated by infection of a local index case on the specified day. The probability of initiation on day *D *is described by the geometric sequence [27]:

(6) $$\mathrm{Pr}\left(D=d\right)={\left(1-p\right)}^{d-1}p.$$

Therefore, the cumulative distribution function of (6) is given by 1-(1-*p*)*^d^*. We calculated the dates at which the cumulative probability first exceeds 50% and 95%, that is, the "average" and "latest plausible" times of local epidemic. The delay in epidemic is derived from the difference in the corresponding dates between the baseline scenario (i.e. without travel restriction) and a scenario in which a fraction of the imported cases were restricted from traveling.

We assess the effectiveness of three different travel restriction policies in four scenarios. In scenarios (i) and (ii), travel restrictions are non-targeted and apply to a fraction of all adults and children, while in scenarios (iii) children or (iv) adults are preferentially restricted from travel. In scenario (i) mixing is assumed to be homogeneous, while in the other scenarios it is heterogeneous, with age assortativity. For selective restrictions (scenarios iii and iv), once the proportion of all travelers that fall into the restricted age group is passed, at 10% for children and 90% for adults, the other age group begins to be targeted.

### Sensitivity analysis

Our results incorporate assortative mixing via the parameter θ, which reflects the proportion of contacts within-group (e.g. child-to-child contacts among the total of contacts made by a single child). If transmission is fully assortative, θ = 1, meaning children contact only children, and adults, only adults. If θ = 0, it implies random or proportionate mixing. We examined the sensitivity of the effectiveness of selective travel restrictions to the assortativity coefficient. While we adopt a published estimate θ= 0.50 at the baseline, we also examined the effectiveness of child-first restriction at θ = 0.10 and 0.90, i.e. plausible but extreme values [13,19]. It should be noted that even our baseline value θ= 0.50 may be regarded as implausibly high, considering the results of social contact survey based on an arbitrary, socially defined "contact" (e.g. [28]), but we adopted 0.50 because this is only the estimate derived from actual epidemic data [13].

## Results

**Figure 1:**
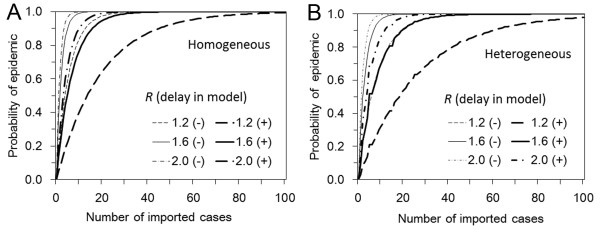
**The probability of epidemic with and without accounting for delay in infection-age among imported cases**. A. The probability of epidemic is calculated as a function of the number of imported cases and the reproduction number (***R***) for a homogeneously mixing population with (+) or without (-) consideration of delay in infection-age among imported cases. B. The case of heterogeneously mixing population.

The probability of an epidemic is shown in Figure 1 as a function of the number of imported cases (ranging from 0 to 100) and the reproduction number with and without accounting for the delay in infection-age among imported cases. In the absence of interventions, the probability of epidemic is greater in homogeneously mixing population (A) than in heterogeneously mixing population (B), but the difference is small and not fully distinguishable unless *R *is small. Accounting for the delay between infection and arrival at the border, the probability of an epidemic increases with the number of imported cases more slowly than when not accounting for infection-age. However, in both Figures 1A and 1B, the probability is in general abruptly elevated with increasing number of imported cases, which echoes the findings in past studies emphasizing that epidemic prevention is unrealistic unless international travel is almost fully restricted [10].

**Figure 2:**
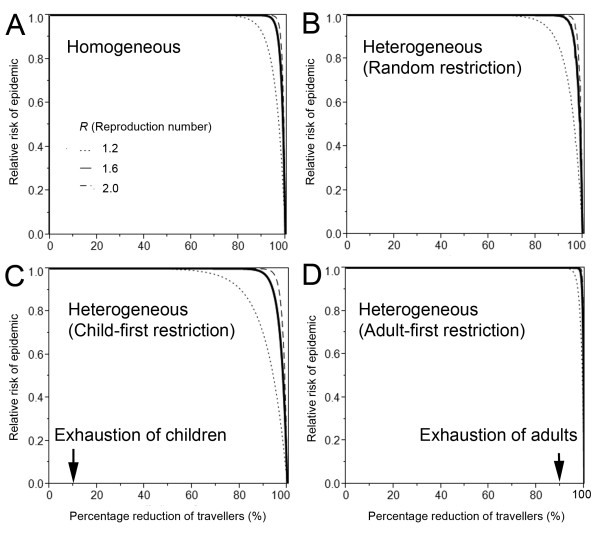
**Relative risk of epidemic by selective and non-selective travel restrictions**. Relative risk of epidemic is shown as a function of the percentage reduction of travelers. In the absence of travel reduction, it is assumed that a total of 500 imported cases arrive in a virgin soil country. Three different reproduction numbers, 1.2 (dotted line), 1.6 (solid line) and 2.0 (dashed line) are considered. A. Non-selective travel restriction in a homogeneously mixing population. B. Non-selective travel restriction in a heterogeneously mixing population. C. Child-first restriction in heterogeneously mixing population. D. Adult-first restriction in heterogeneously mixing population. In C at 10% reduction of travel (specified with arrow), all travels involving children are restricted and the host to restrict travel is switched to adults. Similarly, adult travelers are exhausted at 90% reduction of travel in D.

Given a scenario with a total number of 500 infectious imported cases, Figure 2 compares the effectiveness of different travel restriction policies with different levels of restriction. In all panels A-D, no visible effect is seen as long as movement of 60% or less travelers is restricted. When considering selective travel restrictions in Figures 2C and 2D, both children and adults are exhausted before observing a visible decline in the probability of epidemic. However, slightly fewer restrictions are required under a child-first restriction policy to observe a reduction in the probability of an epidemic.

**Figure 3:**
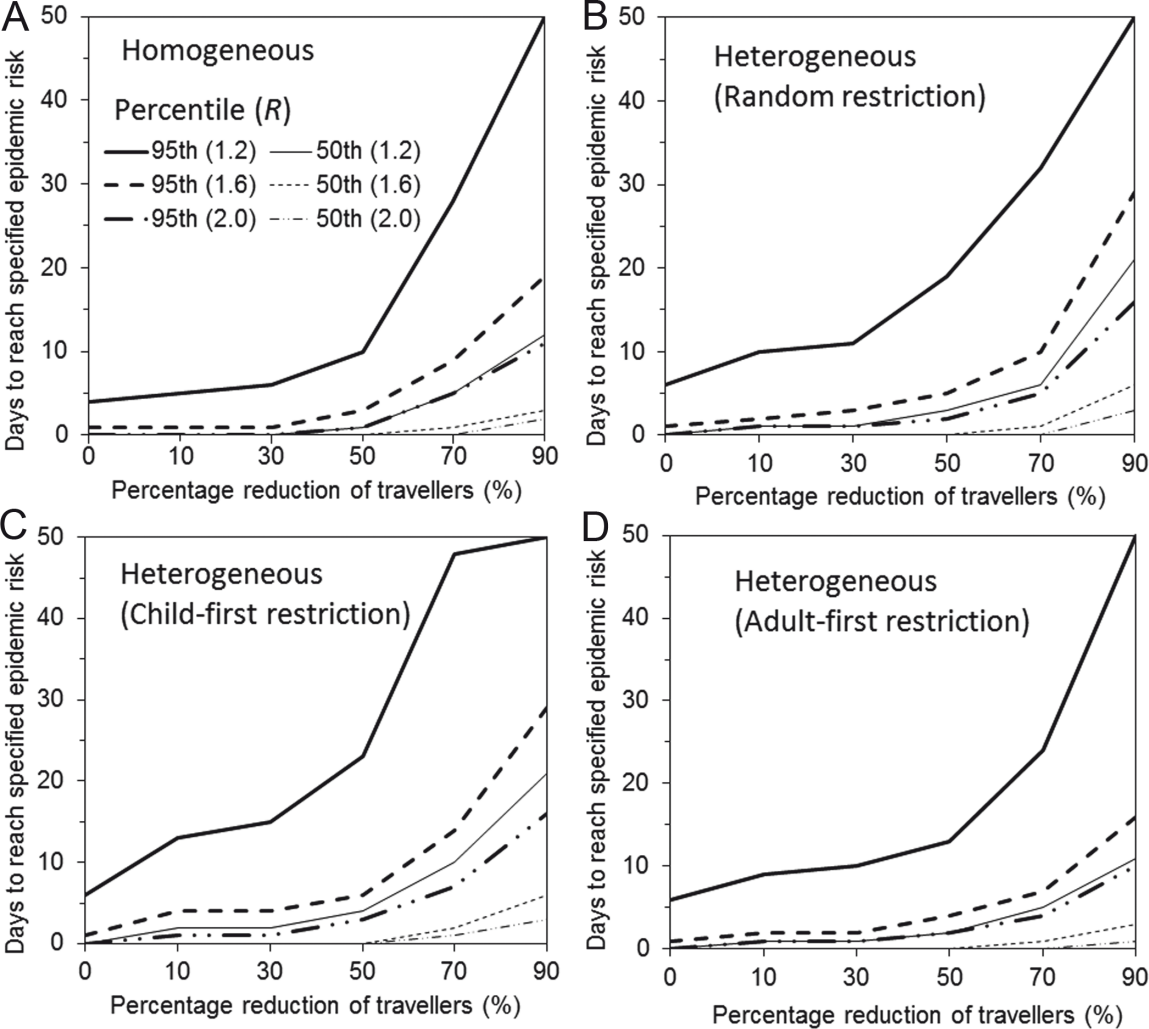
**Delay effect of travel restriction by selective and non-selective travel restrictions**. The first day at which the probability of epidemic reaches 50% or 95% is examined as a function of the percentage reduction of travelers. In the absence of travel reduction, it is assumed that a total of 10 imported cases arrive every day and the importation continues for 50 days (with a total of 500 imported cases). The number of days with travel restriction minus that without restriction gives the delay in epidemic gained by the travel restriction policy. Three different reproduction numbers, 1.2 (solid line), 1.6 (dotted line) and 2.0 (dashed line) are considered. Scenarios A-D are the same as those in Figure 2 (A. homogeneously mixing population; B. random restriction in heterogeneously mixing population; C. child-first restriction and D. adult-first restriction in heterogeneously mixing population).

Based on a scenario with 10 imported cases per day for 50 days, Figure 3 shows the days at which the probability of an epidemic first exceeds pre-specified thresholds (50% or 95%) under different travel restriction policies and as a function of travel volume reduction. Overall, the longest delay (i.e. days at specified travel restriction minus days at 0 percent restriction) is gained by a child-first restriction policy, indicating the importance of accounting for chronological age in considering travel restriction policies. However, even at its most effective, the delay obtained by restricting all children from traveling is shorter than 10 days.

**Figure 4:**
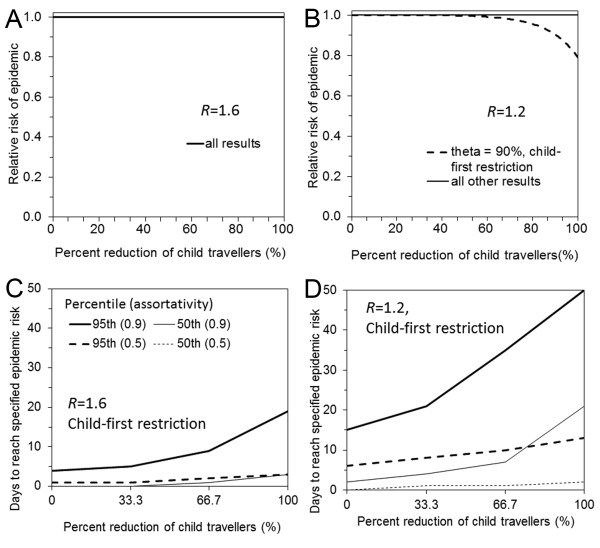
**Sensitivity of the probability of epidemic and days taken to observe epidemic to assortativity coefficient during child-first travel restriction**. Child-first travel restriction is implemented with a total of 500 imported cases (where there are 10 imported cases per day for 50 days). A. Relative risk of epidemic with the reproduction number 1.6 is examined as a function of travel restriction volume. We examine three assortativity coefficient (0.10, 0.50 and 0.90), but the epidemic risk remains consistently 1. B. Relative risk of epidemic with the reproduction number 1.2. Only when the assortativity coefficient (theta = 0.90), a small reduction in the probability of epidemic is observed. C. The first day at which the probability of epidemic reaches 50% or 95% with the reproduction number 1.6. The day with 50% in the case of assortativity coefficient 0.5 is not distinguishable from horizontal axis. D. The first day at which the probability of epidemic reaches 50% or 95% with the reproduction number 1.2.

Figure 4 shows the sensitivity of the probability of epidemic and time delaying effect of child-first travel restrictions to the choice of the assortativity coefficient. Since child travelers are assumed to account for 10% of the total of travelers, selective restriction of child travelers of 100% corresponds to 10% restriction of the total travels. At *R *= 1.6 (and 2.0), there was no visible decline in the probability of an epidemic (A) and the delay until a near certain epidemic (probability>95%) with a full child restriction policy and very high assortativity (θ = 0.90) was shorter than 15 days (C). In a less contagious scenario (*R *= 1.2), only a small decline in the probability of an epidemic was observed with strong assortativity and full child restriction (B). For the range of *R *and θ examined, notable delays in median and 95 percentile points for approximately 19 days and 35 days were observed only with a specific combination of parameters, i.e., *R *= 1.2 and θ = 0.90 (D). Neither a decline in the probability nor a delay to the start of the epidemic was visible with other parameter settings with smaller θ.

## Discussion

The present study examined the effectiveness of selective travel restriction on a heterogeneously mixing population, focusing on full travel restriction among children. The analysis was motivated by two realistic public health issues: (i) children acted as the host maintaining transmission of influenza H1N1-2009 pandemic while adults were relatively less important in transmission [29-32] and (ii) the restriction of all international travel is economically damaging, but restricting child travel (e.g. by cancelling school trips) may be more politically feasible and less damaging to the global and local economies. As was expected, preferentially restricting child travels would be more effective than ignoring age or restricting adults. However, our analysis suggests that such a policy would have marginal public health benefits, only slightly reducing the risk of the outbreak in the short term or delaying the outbreak by a few weeks at best. With all but low transmission potential and low degrees of assortativity, selective travel restrictions offer neither a real reduction in the probability of epidemic nor a substantial delay until it takes off.

The effectiveness of selective travel restrictions has not heretofore attracted scientific attention prior to the H1N1-2009 pandemic, although frequent flyers and their role in facilitating international spread have been closely examined [6]. Many published studies mainly focused on detailed spatial dynamics of transmission in relation to travel restrictions, finding for instance that more than 99% of infected travelers have to refrain from traveling to yield substantial preventive effects [7,9,10]. Given the role of children in propagating H1N1-2009 [13-15] (summarized in Table 2) and considering the potential to put child-only restrictions into practice, we considered age as an important component to determine the effectiveness of travel restrictions and focused on capturing the role of age-dependency on the mechanism of invasion. Our results indicated that while the effectiveness would be marginally sensitive to assortativity, it would not be substantially effective even with high assortivity.

We make several assumptions in this paper and highlight the most important ones. First, we capture the heterogeneities of transmission networks by stratifying over age but not space. As a result, our findings are conservative in the sense that the actual effectiveness of age-structured restrictions may well be slightly greater than those presented here. However, as long as adults can also contribute to generating local child secondary cases, it is natural that adult travel will eventually lead to an epidemic and substantial involvement of adults in transmission would not delay the outbreak substantially.

Second, since our study rests on a simple statistical model, it has a number of further limitations. We describe the generation time distribution via a one-parameter family and it is possible that allowing a second parameter, in making the distribution more realistic, may potentially elevate the probability of extinction [23,33]. Furthermore, it should be remembered that the success of travel restriction depends on travel volume, and this can substantially vary across the world. We examined a plausible number of 10 imported cases per day for the first 50 days through a single port of entry, the scenario most likely to allow effective border closures, but countries with much fewer importations can potentially expect some naturally-occurring delay (e.g. small island nations in Melanesia). Moreover, big countries with multiple ports of entry (e.g. USA and Australia), countries with unmonitored land borders (e.g. those within the Schengen area) and shorter distance of school trip among school-age children as compared to intercontinental travel by adults could prevent the implementation of such a strategy even if it were effective in territories such as Hong Kong. To improve our understanding of this subject further, it might be valuable to account for more detailed heterogeneity (e.g. household and community) and other outcome measurements (e.g. timing and height of epidemic peak), as well as the additional effect of entry screening policies on top of travel restrictions [34], which would help further our understanding of the effectiveness of travel restrictions in realistic settings. Moreover, we should be able to estimate and compare the cost of available policy options. To help relevant policy decisions in the future, we would need more objective epidemiological criteria concerning the severity of disease or an imminent public health risk by elaborating epidemiological details of descriptions given in International Health Regulations [12,35]. The decision may also depend on other available options of control (e.g. if it were realistic to contain an outbreak at local levels, we would not need travel restrictions).

Despite the presence of various realistic features to be explored, the present study has demonstrated that a key policy question can be answered at least qualitatively using a simple statistical model. In conclusion, selective travel restriction of child travelers would have minimal impact on the risk and timeline of an outbreak, even in scenarios most favorable to this strategy. Our findings add to the growing body of evidence that travel restrictions are not viable public health solutions in the face of an emergent influenza pandemic.

## List of abbreviations

*R*: the reproduction number.

## Competing interests

BJC reports receiving research grant funding from MedImmune Inc., a manufacturer of influenza vaccines. All other authors declare that they have no competing interests.

## Authors' contributions

HN conceived of the study and developed methodological ideas. EHYL and HN implemented statistical analyses and drafted the manuscript. JYTW and MSYL contributed to statistical modeling. BJC and ARC gave comments on earlier draft and helped improve the manuscript. All authors read and approved the final manuscript.
